# Time-Varying Nature of Risk Factors for the Longitudinal Development of Disability in Older Adults with Arthritis

**DOI:** 10.2188/jea.JE20090154

**Published:** 2010-11-05

**Authors:** Kuan-Chia Lin, Pau-Chung Chen, J.W.R. Twisk, Hui-Lan Lee, Lin-Yang Chi

**Affiliations:** 1 School of Nursing, National Taipei University of Nursing and Health Sciences, Taiwan; 2 Institute of Occupational Medicine and Industrial Hygiene, College of Public Health, National Taiwan University, Taipei, Taiwan; 3 Department of Epidemiology and Biostatistics, VU Medical Centre, Amsterdam and Department of Health Sciences, Faculty of Earth and Life Sciences, VU University, Amsterdam; 4 Department of Dentistry, National Yang-Ming University, Taiwan

**Keywords:** arthritis, older people, ADL disability, time-varying risk factors, longitudinal study

## Abstract

**Background:**

To investigate changes over time in risk factors for the development of Activities of Daily Living (ADL) disabilities in older adults with arthritis.

**Methods:**

The data were obtained from the Longitudinal Survey of Health and Living Status of the Elderly in Taiwan (1989–1999). The major analytic cohort comprised 977 older adults (458 men and 519 women) with arthritis and without ADL limitation at study baseline. A generalized estimating equations (GEE) model was used to analyze all temporally correlated errors, population-averaged estimates, and longitudinal relationships.

**Results:**

Overall, the cumulative incidence of ADL disability in the analytic cohort was 17.4% during an observation period of 11 years. With respect to baseline risk, ADL disability was associated with older age, presence of comorbid chronic conditions, and poor self-rated health. However, the findings changed after accounting for the time-varying nature of risk factors and the temporal sequence of possible cause-and-effect relationships. In addition to the baseline predictors, a high score on the Center for Epidemiologic Studies Depression Scale, lack of regular exercise, and becoming widowed were associated with an increased risk of ADL disability and a decreased chance of recovery.

**Conclusions:**

An understanding of the time-varying nature of risk factors for the disabling process is essential for the development of effective interventions that aim to maintain functional ability and prevent limitations among older adults with arthritis.

## INTRODUCTION

Arthritis is a chronic condition highly prevalent among older populations.^1^^–^^4^ Aside from its high prevalence, arthritis has a large effect on the health status of elderly populations and, among all chronic conditions, it is the leading cause of long-term disability from both an individual and population perspective.^3^^–^^7^ The necessity of understanding the role and risk of arthritis in developing chronic activities of daily living (ADL) disability is becoming an urgent public health issue.

The development of disability in older adults is complex, as it involves multiple and possibly interrelated disability episodes. It has been noted that disability in older populations is a recurrent rather than an enduring condition, which suggests that intervention to maintain independence after recovery is needed.^8^^–^^11^ The literature on functional limitation in arthritis includes a wealth of cross-sectional studies but few longitudinal studies, and no longitudinal study has investigated an Asian population.^12^^–^^15^

Furthermore, the timing of risk factors may be more important than their mere occurrence during arthritis progression. Unfortunately, previous studies have not specifically examined the time-varying nature of risk factors for the longitudinal development of ADL disability in older adults with arthritis.

**Figure 1.**
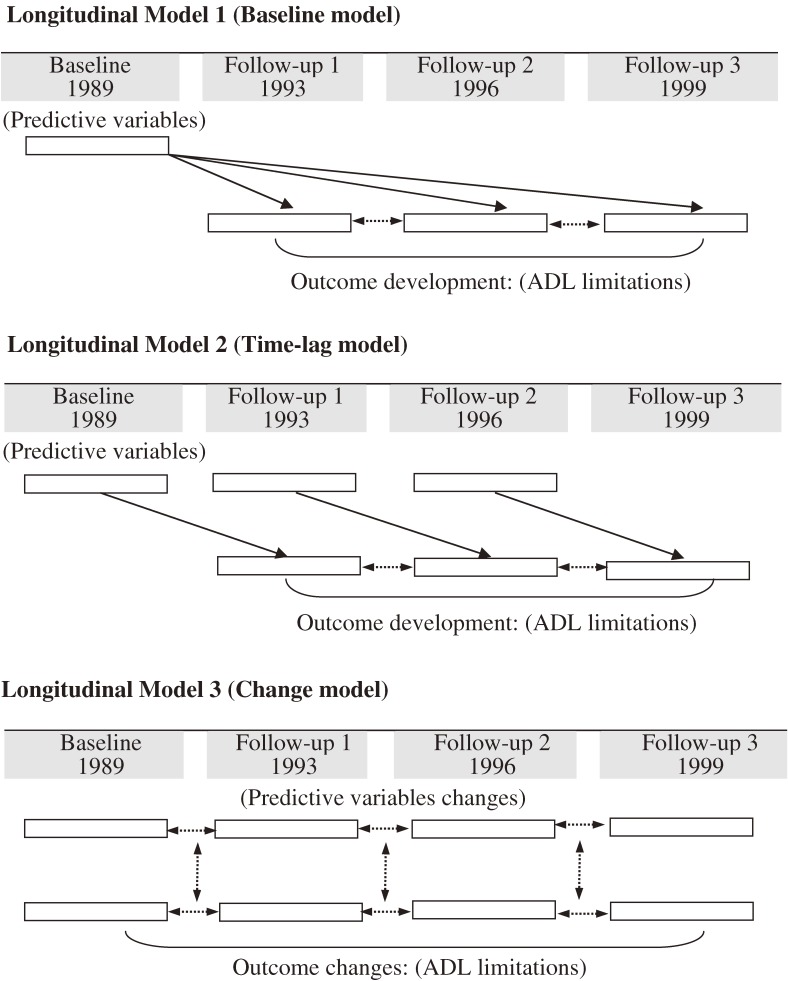
Illustration of 3 longitudinal models used for analysis of risk factors for functional decline in older adults with arthritis. Model 1: Baseline measurements of risk factors were assumed to be related to functional decline reported at 3 follow-up points. Model 2: All risk factors were modeled using data collected at the examination preceding the assessment of the outcome variable (ADL disability). In this model, longitudinal causal relationships between ADL disability and risk factors were analyzed by using all available data, not only with respect to correlation in time sequence, but also with regard to correlation between time-dependent and time-independent variables. Model 3: Changes between 2 consecutive measurements of outcome and predictor variables were studied. We investigated relations between changes in values between different time points, rather than the actual values.

The present study used 3 longitudinal analyses,^16^^–^^19^ including repeated measurements of demographic, lifestyle, and psychological variables and other background data, to assess dynamic risk factors and longitudinal development of ADL disability in older adults with arthritis (Figure 1). The analysis used longitudinal data for a recent 11-year period from the Longitudinal Survey of Health and Living Status of the Elderly in Taiwan.

## METHODS

### Study population

The Longitudinal Survey of Health and Living Status of the Elderly in Taiwan was launched in 1989. It was a cooperative undertaking between the Taiwan Provincial Institute of Family Planning and the Population Studies Center and Institute of Gerontology at the University of Michigan.^20^ An important strength of this survey is that it represents the entire elderly population of Taiwan. The design of the original study is described briefly here and in detail elsewhere.^20^^–^^23^

Overall, a total of 4049 subjects completed interviews for the baseline study (1989), which corresponded to a response rate of 91.8%. Proxy interviews were needed for only 189 cases, representing 4.4% of all completed interviews and 4.1% of the total sample. Proxies provided information only on matters that could be observed. Their reports appeared to be reliable.^20^^–^^23^ Three follow-up interviews (in 1993, 1996, and 1999) were the longitudinal components of the survey. The number of cumulative deaths was 582 in 1993, 1047 in 1996, and 1486 in 1999. Response rates for the 3 follow-up surveys were 91.0%, 88.9%, and 90.1%, respectively.

### Arthritis classification

A subject was classified as having arthritis if they answered affirmatively to the question, “Have you ever had, or has a doctor ever told you that you have, arthritis or rheumatism?” Self-reported history of arthritis is relevant from a public policy perspective because many persons with arthritis do not consult a health care provider for treatment.^24^ In addition, subjects with arthritis were asked another question, “How much inconvenience has arthritis caused in your daily living?” The response options included, on an ordinal scale: “no inconvenience at all,” “some inconvenience,” and “a lot of inconvenience.” The latter 2 responses were defined as “self-perceived inconvenience caused by arthritis.”

### Disability outcomes

All 4 interviews included identical sets of questions regarding self-reported information on disability status. The modified Katz ADL Scale^25^ was used to assess self-reported ability to perform 6 basic activities without help: bathing, dressing, walking across a room, transferring from a bed to a chair, eating, and toileting. For each set of questions, a score corresponding to the number of items with disability was calculated. Thus, higher scores indicated greater limitation in physical function, and a score of 0 indicated no difficulty in all items. If there was a missing response to any item, that set of questions was treated as missing.

### Assessment of other covariates

Other variables included in the analysis were structured questions about sociodemographic characteristics, health, psychological variables, and lifestyle. Sociodemographic characteristics included age (in years), sex, education (years of schooling completed), and marital status (married, widowed, and other). Health and psychological variables included body mass index (BMI), and a summary measure of chronic health conditions was calculated using a diagnosis checklist. Self-rated health was assessed using a single item, “Regarding your state of health, do you feel it is excellent [coded 1], good, average, not so good, or very poor [coded 5].” Thus, higher scores indicated a perception of poorer health. In identifying trajectory stability, 5-category self-rated health was then simplified into “good” (excellent, good, or average) and “poor” (not so good or very poor). The presence of depressive symptoms was then evaluated by using an abbreviated 10-item version of the Center for Epidemiological Studies–Depression Scale (CES-D).^26^ Lifestyle measures assessed included personal habits such as betel chewing, alcohol consumption, cigarette smoking, and routine exercise. These habits were evaluated by using the questions, “Do you [description of habit]?” and “Did you ever [description of habit]?”

### Statistical analysis

The progression of study variables was described at baseline and during follow-up, and is summarized as the mean and standard deviation for continuous variables and as proportions for categorical variables. The generalized estimating equation (GEE) method was used to analyze repeated measurements.^16^ The GEE method extends standard regression analysis and accounts for the correlation between repeated measurements. Three longitudinal models were used in this study (Figure 1). In longitudinal model 1 (baseline model), all risk factors were measured at baseline. These baseline measurements were hypothesized to be related to ADL disability reported at the 3 follow-up points. In longitudinal model 2 (time-lag model), all risk factors were modeled using data from the examination that preceded measurement of the outcome variable (ADL disability). In this model, longitudinal causal relationships between ADL disability and risk factors were analyzed by using all available data. Correlation in time sequence was investigated, as was correlation between time-dependent and time-independent variables. Therefore, the time-lag model yielded a better understanding than model 1 of the specific time-varying nature of exposures. In addition, the time-lag model accounts for the temporal sequence in a possible cause-and-effect relationship. Finally, in order to assess changes occurring between 2 consecutive measurements of both the outcome and predictor variables, longitudinal model 3 (change model) analyzed associations between changes in values, rather than those between the actual values. In model 1 and model 2, scores for ADL disability were dichotomized according to whether there was a disability in 1 or more items (score = 1) or not (score = 0). Then, the model with a dichotomous outcome variable was analyzed using (longitudinal) logistic regression, and odds ratios (ORs) were calculated. However, model 3 was introduced to analyze changes in ADL numbers between different time-points. In all analyses, a *P* value <0.05 was considered to indicate statistical significance. All longitudinal analyses were performed with the statistical package SAS version 9.13 (SAS Institute Inc, Cary, NC, USA).

## RESULTS

Table 1 shows information on respondents and response rates for each study year. As the table shows, the cumulative number of deaths was 582 in 1993, 1047 in 1996, and 1486 in 1999. Response rates for the 4 surveys were 91.8%, 91.0%, 88.9%, and 90.1%, respectively. One particularly attractive feature was the extraordinarily high response rate that was achieved from the baseline study to follow-ups, so the loss to follow-up bias would be not to an important degree.^20^^–^^23^ Because the current study was principally interested in risk factors for the development of disability in older adults with arthritis, the eligible study population was initially defined as adults older than 60 years at baseline who were free of any form of ADL disability. A total of 3353 subjects were thus included in the study cohort. Then, the study cohort was then divided into 2 analytic cohorts composed of 2376 subjects (1151 men and 865 women) without arthritis at baseline and 977 subjects (458 men and 519 women) with arthritis at baseline (the major analytic cohort for this study; Table 2).

**Table 1.** Number of respondents and response rate for each study year of the Longitudinal Survey of Health and Living Status of the Elderly in Taiwan

	Calendar year			
	
	1989	1993	1996	1999
Probability sample (*n*)	4412			
Completed cohort (*n*)	4049	3155	2669	2310
Death (*n*)	—	582	1047	1486
Loss to follow-up (*n*)	363	312	333	253
Respondent rate (%)	91.8	91.0	88.9	90.1

**Table 2.** Physical function, demographic, psychological, and lifestyle variables, and chronic health conditions of subjects at baseline (1989) and follow-up (1993–1999) examinations

	Calendar year			
	
	1989	1993	1996	1999
Study cohort^a^ (*n*)	3353	2712	2321	2017
Sex (Male/Female)	1969/1384	1569/1143	1333/988	1136/881
Age, yrs (mean ± SD)	67.56 ± 6.12			

Analytic cohort 1^b^ (*n*)	2376			
Sex (Male/Female)	1511/865			
Age, yrs (mean ± SD)	67.52 ± 6.17			
Education, yrs (mean ± SD)	4.25 ± 4.70			
ADL limitation				
Moderate (1–2 ADL limitations)	—	1.5%	2.3%	2.9%
Severe (3+ ADL limitations)	—	3.8%	5.3%	8.8%

Analytic cohort 2^c^ (*n*)	977	778	666	588
Sex (Male/Female)	458/519	359/419	306/360	258/330
Age, yrs (mean ± SD in yrs)	67.68 ± 6.01			
Education, yrs (mean ± SD in yrs)	3.86 ± 4.56			
ADL limitation				
Moderate (1–2 ADL limitations)	—	3.1%	2.9%	3.1%
Severe (3+ ADL limitations)	—	5.8%	9.5%	14.3%
No. of chronic health conditions (mean ± SD)	2.24 ± 1.73	2.25 ± 1.65	2.32 ± 1.76	2.76 ± 1.90
CES-D score (mean ± SD)	7.50 ± 5.49	7.56 ± 6.27	7.55 ± 6.16	7.52 ± 6.87
Self-perceived health (mean ± SD)	5.46 ± 1.30	5.37 ± 1.41	4.78 ± 1.26	5.91 ± 1.37
Routine physical exercise	20.3%	34.1%	35.4%	34.0%
Body mass index (kg/m^2^)	23.60 ± 3.83	23.35 ± 3.40	22.82 ± 3.44	22.75 ± 3.42
Smoking (current)	29.8%	23.9%	23.2%	20.1%
Alcohol consumption (current)	18.7%	16.5%	16.4%	20.5%
Betel chewing (current)	5.1%	4.9%	3.8%	4.1%
Being widowed	35.7%	38.7%	46.1%	58.1%

^a^Subjects without ADL limitation at baseline.

^b^Subjects without arthritis or ADL limitation at baseline.

^c^Subjects with arthritis and without ADL limitation at baseline.

Abbreviations: SD, Standard Deviation; ADL, Activities of Daily Living; CES-D, Center for Epidemiological Studies–Depression Scale.

Table 2 shows information on the subjects’ physical function, demographic, psychological, and lifestyle characteristics, and chronic health conditions at baseline, in 1989, and at follow-up in 1993, 1996, and 1999. Overall, the cumulative incidence of ADL disability during the 11-year follow-up period was 2.9% in analytic cohort 1 and 3.1% in analytic cohort 2, among those with moderate disability (1–2 ADL limitations), and 8.8% and 14.3%, respectively, among those with severe disability (3+ ADL limitations).

**Figure 2.**
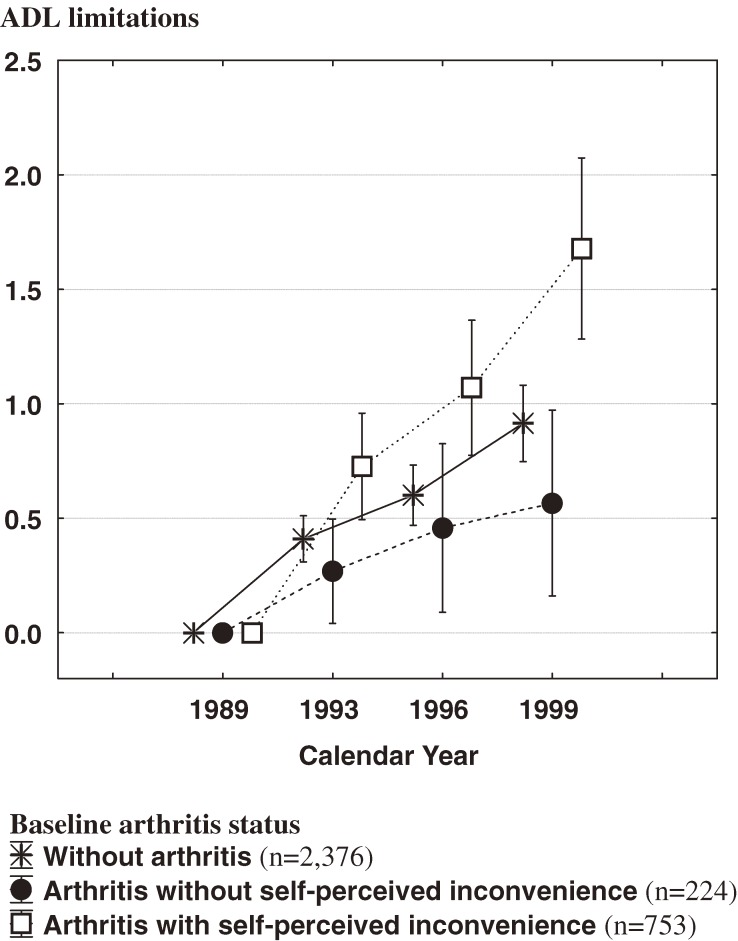
Changes in mean activities of daily living (ADL) score during the 11-year follow-up period, by baseline arthritis status (higher scores indicate a greater number of ADL limitations; a score of 0 indicates no difficulty on any item).

At baseline, the proportion of subjects with self-perceived inconvenience was 77.1% (753/977) among those with arthritis. Figure 2 displays longitudinal changes in mean ADL and its 95% confidence interval from baseline to 1999. There was an increasing trend in ADL limitations for all 3 groups from the study cohort. In addition, the results indicate that the average rate of increase during the follow-up period was highest among subjects with arthritis who reported inconvenience due to the condition. By contrast, the lowest average rate was found among subjects with arthritis and no self-perceived inconvenience.

To determine the strongest predictors of development of ADL disability in older adults with arthritis, the relative effects were estimated by using adjusted ORs and regression coefficients, after controlling for demographic differences, chronic health conditions, health behaviors, psychological factors, and other study variables (Table 3).

**Table 3.** Odds ratios^a^ (ORs, model 1 and model 2), regression coefficients^a^ (model 3), and 95% confidence intervals (CIs) in univariate and multivariate analysis of risk factors for ADL disability in older adults with arthritis, at baseline (1989) and follow-up (1993, 1996, and 1999)

Parameters	Longitudinal model 1 (Baseline model)				Longitudinal model 2 (Time-lag model)				Longitudinal model 3 (Changes model)			
		
	Univariate analysis		Multivariate analysis		Univariate analysis		Multivariate analysis		Univariate analysis		Multivariate analysis	
					
	OR	95% CI	OR	95% CI	OR	95% CI	OR	95% CI	β	95% CI	β	95% CI
Age, yrs	1.10	1.07 to 1.12	1.09	1.07 to 1.13	1.13	1.10 to 1.15	1.09	1.05 to 1.12	0.09	0.07 to 0.11	0.08	0.07 to 0.12
Sex (male vs female)	0.72	0.52 to 0.98	NS	NS	0.72	0.52 to 0.98	NS	NS	−0.14	−0.38 to 0.13	NS	NS
Self-perceived inconvenience ​ caused by arthritis (yes vs no)	2.46	1.58 to 3.82	NS	NS	2.65	1.63 to 4.31	NS	NS	0.75	0.38 to 1.13	0.59	0.28 to 0.97
No. of chronic health conditions	1.21	1.10 to 1.32	1.13	1.02 to 1.25	1.26	1.18 to 1.35	1.15	1.04 to 1.26	0.11	0.08 to 0.15	0.08	0.04 to 0.28
Self-rated health^b^	1.29	1.13 to 1.45	1.21	1.05 to 1.39	2.10	1.82 to 2.43	1.57	1.27 to 1.94	0.22	0.15 to 0.27	0.15	0.09 to 0.31
Routine exercise (yes vs no)	0.85	0.57 to 1.27	NS	NS	0.23	0.17 to 0.32	0.40	0.26 to 0.62	−1.33	−1.07 to −1.47	−1.24	−1.05 to −1.41
CES-D score^c^	1.05	1.02 to 1.08	NS	NS	1.06	1.05 to 1.07	1.07	1.04 to 1.10	0.06	0.04 to 0.08	0.06	0.05 to 0.07
Being widowed (yes vs no)	1.88	1.38 to 2.63	NS	NS	2.43	1.81 to 3.22	1.42	1.13 to 2.01	0.45	0.21 to 0.68	0.36	0.29 to 0.83

^a^The working correlation matrix is autoregressive.

^b^Higher scores indicate poorer self-rated health status; ^c^Higher scores indicate a greater number of depressive symptoms.

Other study covariates that were not significant in multivariate analysis were included, namely, education level, body mass index, smoking, alcohol consumption, and betel chewing.

NS: not significant.

Abbreviations: ADL, activities of daily living; CES-D, Center for Epidemiological Studies–Depression Scale.

In model 1 (baseline model), significant risk factors for longitudinal development of ADL disability at baseline were age (adjusted OR, 1.09; 95% CI, 1.07–1.13), number of chronic health conditions (1.13, 1.02–1.25), and poor self-rated health (1.21, 1.05–1.39).

In model 2 (time-lag model), all risk factors were modeled using data from the examination that preceded measurement of the outcome variable (ADL disability). Thus, in the analysis of change during the 11-year follow-up period, the temporal sequence of possible cause-and-effect relationships between risk factors and ADL disability could be evaluated. Development of ADL disability was significantly associated with age (adjusted OR, 1.09; 95% CI, 1.05–1.12), number of chronic health conditions (1.15, 1.04–1.26), poor self-rated health (1.57, 1.27–1.94), CES-D score (1.07, 1.04–1.10), with regular exercise (0.40, 0.26–0.62), and being widowed (1.42, 1.13–2.01).

In model 3 (change model), changes between 2 consecutive measurements of ADL disability were significantly associated with age (adjusted β = 0.08; 95% CI, 0.07–0.12), self-perceived inconvenience caused by arthritis (0.59, 0.28–0.97), number of chronic health conditions (0.08, 0.04–0.18), poor self-rated health (1.57, 1.27–1.94), CES-D score (0.06, 0.05–0.07), regular exercise (−1.24, −1.05 to −1.41), and being widowed (0.36, 0.29–0.83). Finally, in all models, there was no particular “period” effect (eg, in the baseline risk model, calendar year 1999 vs 1993: OR = 0.73, 95% CI 0.51–1.15; calendar year 1996 vs 1993: OR = 0.83, 95% CI 0.62–1.10).

## DISCUSSION

Given the worldwide increase in the numbers of older adults with arthritis, understanding the role of arthritis in the development of ADL disability is becoming an urgent public health issue. Risk factors for the onset of ADL limitations in older adults with arthritis have been discussed previously.^3^^,^^15^^,^^27^^–^^29^ Our study presents evidence of the time-varying nature of these risk factors on the development of ADL disability in older adults with arthritis. The advantages of our analysis as compared with traditional techniques are: 1) all longitudinal data are used to estimate one regression coefficient indicating the overall relationship, 2) all relationships can be estimated after correction for both time-dependent and time-independent covariates, and 3) when a GEE model is used to calculate the parameters of these 2 models, it is possible that different subjects have a different number of repeated measurements. Hence, with respect to baseline risk, the results indicate that older age, presence of comorbid chronic conditions, and poor self-rated health were associated with ADL disability over a period of 11 years in older adults. However, the findings changed after taking into account the time-varying nature of risk factors and possible cause-and-effect relationships. In addition to the baseline risk factors, higher CES-D score, lack of regular exercise, and being widowed were also significantly associated with an increased risk of becoming ADL-disabled during follow-up.

It has been reported that the onset of ADL disability is a complex process, and that medical, demographic, psychological, and behavioral triggers are all important.^30^^,^^31^ Our observation that older age, presence of comorbid chronic conditions, and poor self-rated health are associated subsequent ADL deterioration is in agreement with previous research.^3^^,^^15^^,^^28^^,^^31^^–^^38^ Moreover, our data indicate that these 3 factors and their timing have different epidemiologic roles in the risk of arthritis. As noted above, we found that older age, presence of comorbid chronic conditions, and poor self-rated health were the only predictors of ADL disability at baseline. However, the time-varying nature of these 3 factors was also important in the longitudinal development of ADL disability among older adults with arthritis. It may be that older age, comorbid chronic conditions, and poor self-rated health are the initial biological conditions that increase the subsequent risk of ADL disability, and that the risks posed by these factors are proportional to the length of exposure to, or increase in, these 3 factors. Our focus on the experience of an arthritis cohort makes these findings relevant to the design of public health interventions and prevention programs that aim to maintain and improve function among older adults with this condition.

In addition to older age, presence of comorbid chronic conditions, and poor self-rated health, our analysis of longitudinal time-varying relationships indicates that CES-D score, lack of regular exercise, and being widowed could predict the development of ADL disability during follow-up, which suggests the importance of the temporal sequence and time-varying nature of risk factors in the relationships between arthritis and subsequent ADL disability. This was consistent with evidence indicating that regular exercise reduces the incidence of ADL disability in older adults with knee osteoarthritis.^15^^,^^34^^,^^35^ Exercise may be an effective strategy for preventing ADL disability and, consequently, may extend the period of autonomy in older adults. Regarding depressive symptoms, the potential advantages of including a psychoeducational intervention in treatment options for arthritis are becoming increasingly recognized.^33^ Studies have also shown that arthritis patient education programs can be useful for enhancing self-care management techniques and improving physical and psychological health outcomes.^37^^,^^38^ Furthermore, it has been reported that becoming widowed and the duration of widowhood are important determinants for the onset of late-life disability in different disability domains.^39^^,^^40^ Previous study results have also highlighted the importance of focusing not only on the short-term effects of socioeconomic factors, but also on the possibility of long-term effects on disability in older adults.^41^ From an epidemiologic perspective, our results indicate that lack of regular exercise, depressive symptoms, and becoming widowed are associated with an increased likelihood of ADL disability and a decreased chance of recovery in older populations with arthritis. Moreover, the combination of baseline and time-varying risk factors may increase the future risk of ADL disability.

It is interesting to note that subjects with arthritis who had no self-perceived inconvenience had a lower incidence of ADL disability than did subjects without arthritis. In fact, in our crude analysis, self-perceived inconvenience caused by arthritis was a significant factor in all longitudinal models, particularly with respect to between-examination changes in ADL limitations. However, the relationship became insignificant in the other 2 longitudinal models, after adjustment for age, comorbid chronic conditions, poor self-rated health, marital status, and CES-D score. Interestingly, changes in ADL numbers between different time-points were related. The change model allows separation between the within-person relationship (longitudinal) and the between-person relationships (cross-sectional). In view of the fact that the other 2 models showed no independent relation, it is possible that, in subjects with self-perceived inconvenience who developed ADL disability, longitudinal within-person relationships will be overruled by the between-person relations when the variation in actual values between subjects exceeds the change over time within subjects. In addition, the results also suggest the presence of interrelationships between self-perceived inconvenience caused by arthritis and other risk factors for ADL disability. Also, the disabilities of adults with arthritis tend to accumulate gradually rather than occurring simultaneously.^42^ It has been reported that, during the course of arthritis, patients can learn to adjust to their condition and its consequences, and are thus able to maintain a normal stress level.^43^ Other studies have noted substantial intraindividual variation, which may be due to differences in disease-related factors such as joint tenderness, pain, and disability.^9^^–^^11^^,^^44^ A previous study reported that a significant relationship exists between arthritis onset and worsening pain, resulting in the development of activity limitation.^32^ However, our analysis did not control for chronic pain conditions due to the lack of such information in the present cohort data. Chronic pain is probably associated not with only arthritis, but also self-rated health, motivation for and compliance with regular exercise, and depressive mood. Therefore, a better understanding of how chronic pain affects the interrelationships between self-perceived inconvenience caused by arthritis and other risk factors in the development of disability is essential if effective prevention and intervention programs are to be developed. That is, we need to recognize how different trajectories in self-perceived inconvenience caused by arthritis are related to the disabling process in adults with different demographic, biological, and psychological characteristics.

This report suffers from a number of limitations, which we will briefly discuss. First, although arthritis is one of the most important causes of disability in elderly individuals, especially impairment of the joints of the lower extremities, rheumatoid arthritis (RA) could not be distinguished from osteoarthritis (OA) in our analysis. Inconvenience and ADL disability depend on whether an impairment originates in the lower or upper extremities. Therefore, arthritis may lead to severe and even total disability in an individual, especially when it is associated with other comorbidities (eg, dementia).^45^^,^^46^ However, from a population perspective, the combining of RA and OA is not of great importance. Nevertheless, additional studies are needed to further examine the time-varying nature of risk factors in the disabling process of older adults with rheumatoid arthritis. A second limitation of this study is that the findings do not consider the influence of additional factors (eg, specific cognitive impairments or environmental factors) known to influence the development of disability. Despite the absence of these factors in our analysis, the current exploratory analyses do provide sufficient evidence to discuss the importance of the time-varying nature of risk factors in the longitudinal development of disability in an older population. Future studies will likely examine a more comprehensive set of risk factors and their associations with different functional outcomes. Finally, our study results are limited to an older Chinese population, so studies in other ethnic populations are necessary.

In conclusion, this research has revealed longitudinal relations between risk factors and the development of ADL disability in older adults with arthritis. Older age, presence of comorbid chronic conditions, and poor self-rated health were the most important risk factors at baseline and need to be carefully monitored. In addition, lack of regular exercise, depressive symptoms, and becoming widowed may increase the risk of ADL disability and decrease the chance of recovery in older adults with arthritis. An understanding of the time-varying nature of risk factors in the disabling process is essential to develop effective interventions to help maintain functional ability and prevent limitations among older adults with arthritis.
